# Severe Tetanus in an Older Adult Without an Identifiable Portal of Entry: A Case Report

**DOI:** 10.1155/crdi/3340319

**Published:** 2026-09-29

**Authors:** Yanwei Rao, Wei He, Yang Yu, Yicheng Wang, Yuan Yuan, Yongjie Wang

**Affiliations:** ^1^ Department of Critical Care Medicine, Jilin Province People’s Hospital, Changchun 130021, China, jlsyy.net; ^2^ Jilin Province Clinical Medical Research Center for Emergency & Critical Care Medicine, Changchun 130021, China; ^3^ Department of Hepatobiliary and Pancreatic Surgery, Jilin Province People’s Hospital, Changchun 130021, China, jlsyy.net; ^4^ Department of Urology, Jilin Province People’s Hospital, Changchun 130021, China, jlsyy.net; ^5^ Changchun University of Chinese Medicine, Changchun 130021, China, ccucm.edu.cn

**Keywords:** aged, case report, diagnosis, occult wounds, tetanus

## Abstract

Tetanus remains a significant health concern, particularly in older adults with waning immunity. The diagnosis is typically clinical, based on characteristic features such as trismus, muscle rigidity, and stimulus‐triggered spasms. However, diagnostic challenges arise when tetanus presents without an identifiable acute wound or with an occult portal of entry, as it may be misattributed to more common neurological conditions such as stroke or encephalitis. We report a 65‐year‐old Chinese male who presented with 15‐day cough and sputum, followed by acute trismus and dysphagia. Initial evaluation at a local hospital suggested cerebral infarction, but treatment with neuroprotective agents yielded no improvement. The patient was transferred to a tertiary hospital, where he developed generalized spasms and respiratory distress requiring mechanical ventilation. Extensive neurological investigation revealed only chronic ischemic changes; encephalitis was considered but not confirmed. Subsequent consultation identified characteristic features including trismus, risus sardonicus, opisthotonos, stimulus‐triggered spasms, and preserved consciousness during spasms, leading to diagnosis of severe tetanus (Ablett Grade IV, based on severe generalized spasms, respiratory failure requiring mechanical ventilation, and autonomic dysfunction requiring vasopressor support). No acute wound was identified on full‐body examination; only remote facial burns and scarring from > 20 years prior was noted. The patient received human tetanus immunoglobulin, intensive sedation and analgesia, mechanical ventilation, and supportive care. After 28 ICU days, the patient was successfully weaned from ventilation, achieved complete spasm resolution, and was transferred to a general ward for rehabilitation. This case demonstrates that severe generalized tetanus should remain in the differential diagnosis of older adults presenting with acute trismus, dysphagia, muscle rigidity, or stimulus‐triggered spasms, even when recent trauma is not recalled and no active wound is visible. Clinicians should obtain a thorough exposure history, perform repeated full‐body examinations, and initiate timely antitoxin administration and ICU‐based supportive care. The absence of an identifiable portal of entry should not delay diagnosis or treatment.

## 1. Introduction

Tetanus, caused by the neurotoxin tetanospasmin produced by *Clostridium tetani*, remains a significant global health concern despite the availability of effective prevention through vaccination and appropriate wound care [1–3]. The disease is particularly prevalent in regions with inadequate vaccination coverage and in older adults with waning immunity [3–5]. The pathogenesis begins with contamination of wounds by *C. tetani* spores, which germinate in anaerobic conditions and produce tetanospasmin. The toxin undergoes retrograde axonal transport to the central nervous system, where it blocks inhibitory neurotransmission by cleaving synaptobrevin, leading to uncontrolled motor neuron excitation, profound muscle rigidity, autonomic dysfunction, and life‐threatening spasms. The diagnosis of tetanus is typically clinical, relying on characteristic features such as trismus (lockjaw), muscular rigidity, and stimulus‐triggered painful spasms, including risus sardonicus and opisthotonos [1, 3, 5–7]. Classically, tetanus follows an acute injury that provides a portal of entry for the bacterium, with symptoms developing within days to weeks [8]. However, the disease presents formidable diagnostic challenges when an acute, identifiable wound is absent or when the incubation period is prolonged. In such atypical presentations, tetanus may be misattributed to more common neurological conditions such as cerebral infarction, encephalitis, or other causes of altered mental status and spasticity, particularly in elderly patients where cerebrovascular disease is prevalent [6]. The differential diagnosis of acute trismus and dysphagia in older adults is broad and includes not only stroke and encephalitis but also drug‐induced dystonia, dental or deep neck infections, temporomandibular disorders, hypocalcemia, neuroleptic malignant syndrome, and toxicologic causes. This case illustrates the diagnostic challenges and clinical reasoning required when tetanus presents without an obvious portal of entry, emphasizing the importance of maintaining a high index of suspicion in appropriate clinical contexts.

## 2. Case Presentation

### 2.1. Patient Information and Initial Presentation

On May 12, 2025, a 65‐year‐old Chinese male presented to a local hospital with a 15‐day history of cough and sputum production. His past medical history was notable for cerebral thrombosis, which was managed without residual deficits, and facial burns and trauma with scarring sustained more than 20 years prior. The patient developed acute trismus and inability to eat or drink. Initial evaluation suggested cerebral infarction, and treatment with neuroprotective agents and conservative stroke management was initiated. However, no clinical improvement was observed.

### 2.2. Hospital Course and Diagnostic Evaluation

On May 18, 2025, the patient was transferred to a tertiary hospital for further evaluation. Admission computed tomography (CT) of the head revealed multiple lacunar infarcts, leukoaraiosis, and age‐related brain changes. Chest CT showed mild left‐sided pneumonia, bilateral pulmonary nodules, and left pleural effusion. Following admission, the patient developed generalized spasms and respiratory distress, necessitating endotracheal intubation and mechanical ventilation.

Brain magnetic resonance imaging (MRI) confirmed multiple lacunar infarcts, leukoaraiosis, enlarged Virchow–Robin spaces in the left semiovale center, bilateral frontotemporal subdural hygromas, and severe pansinusitis. Investigations ruled out acute cerebrovascular events. Encephalitis was considered but required further workup.

For further diagnosis and treatment, the patient’s family requested a consultation from our hospital’s critical care team. During the consultation, the patient exhibited characteristic findings including trismus (lockjaw), generalized muscle rigidity, painful stimulus‐induced spasms, opisthotonos, and risus sardonicus (a wry, sardonic smile). Importantly, the patient remained conscious during spasms, with consciousness returning to baseline between episodes. Based on the above symptoms, the patient was diagnosed with tetanus (Ablett Grade IV, based on severe generalized spasms, respiratory failure requiring mechanical ventilation, and autonomic dysfunction requiring vasopressor support). The patient was transferred to our intensive care unit (ICU) for subsequent management.

### 2.3. ICU Admission and Initial Assessment

Upon admission to the ICU, the patient was diagnosed with tetanus (Ablett Grade IV), pneumonia, lacunar infarcts, and post‐tracheostomy status. A comprehensive physical examination, performed by a senior attending physician within 12 h of ICU admission, revealed no active wounds, ulcers, or recent injuries. The examination included detailed inspection of the scalp, oral cavity, nail beds, interdigital spaces, soles, and perianal region. The only identifiable skin abnormality was healed facial scarring from burns sustained more than 20 years prior. No other potential portals of entry were identified on examination of the soles, toes, nail folds, oral cavity, perianal region, or other anatomical sites. The patient’s tetanus vaccination history was unavailable, as the patient could not recall previous immunizations.

During the early ICU course, the patient developed significant autonomic dysfunction, manifested by marked blood pressure lability with systolic blood pressure fluctuating between 80 and 190 mmHg, episodes of severe tachycardia (heart rate 120–150 beats/min), and diaphoresis. These autonomic fluctuations were temporally associated with spontaneous spasms as well as external stimuli such as suctioning and repositioning. Norepinephrine continuous intravenous infusion was initiated on ICU Day 3 at a dose of 0.05 μg/kg/min, titrated up to a maximum of 0.3 μg/kg/min, and successfully weaned off by ICU Day 13. No arrhythmias requiring intervention were observed. This autonomic instability, in conjunction with severe generalized spasms and respiratory failure, supported the Ablett Grade IV classification.

### 2.4. Social and Environmental History

The patient was a retired factory worker residing in a rural area of Jilin Province. He reported no recent farming and gardening or prolonged outdoor activities. Nevertheless, his preretirement occupational history included extensive agricultural work and manual labor with regular soil contact, which constituted a plausible historical route of exposure to *C. tetani* spores. This exposure profile is consistent with the established epidemiological pattern of tetanus. He also denied any recent travel history, animal bites, or documented penetrating injuries.

### 2.5. Treatment and ICU Management

Upon admission to the ICU, the patient was immediately initiated on a comprehensive tetanus management protocol. On the day of admission (ICU Day 1), Human Tetanus Immunoglobulin (HTIG, 3000 IU) was administered intravenously to neutralize circulating unbound tetanospasmin. Concurrently, antimicrobial therapy with intravenous metronidazole (500 mg every 8 h) was initiated on ICU Day 1 and continued for a total of 10 days to eliminate vegetative *C. tetani* organisms. As no identifiable wound was detected on thorough full‐body examination, wound debridement was not performed.

Respiratory support was instituted with the patient remaining intubated and mechanically ventilated throughout the acute phase. Initial ventilator settings were configured as follows: Mode VC‐SIMV, Tidal volume 500 mL, Respiratory rate 14 breaths/min, Pressure support 12 cmH_2_O, PEEP 5 cmH_2_O, and FiO_2_ 50%. These settings were subsequently adjusted based on arterial blood gas analysis and clinical response.

Upon ICU admission, the patient exhibited a high frequency of generalized spasms, with up to 16 episodes occurring within a 24‐h period. To achieve adequate spasm control, continuous intravenous infusions of multiple sedative and analgesic agents were initiated, including remifentanil, midazolam, propofol, a hibernation mixture, and dexmedetomidine. Infusion rates were carefully titrated based on spasm frequency and clinical response and were progressively reduced as the patient’s condition improved. Notably, a transient exacerbation of spasms occurred between ICU Days 9 and 12, peaking at 26 episodes per day, which necessitated temporary upward adjustments to sedative and analgesic infusion rates. Thereafter, spasm frequency progressively declined to 20 episodes/day by Day 13, and further to 6 episodes/day by Day 21, allowing continued weaning of sedation. Autonomic dysfunction was managed with continuous norepinephrine infusion as described above, alongside maintaining adequate sedation and analgesia to minimize sympathetic surge. Blood pressure, heart rate, and respiratory parameters were continuously monitored, with hourly vital sign documentation. Fluid resuscitation was guided by dynamic parameters including pulse pressure variation and stroke volume variation, aiming to maintain euvolemia while avoiding fluid overload.

Fluid and electrolyte management was guided by the recognition that severe and frequent muscle spasms, profuse diaphoresis, increased metabolic demand, and potential ventilatory irregularities placed the patient at significant risk of metabolic derangement. To prevent metabolic derangement, arterial blood gases and serum electrolytes (sodium, potassium, chloride, calcium, and magnesium) were assessed every 6–8 h, with deficits promptly corrected to maintain physiological homeostasis.

Nutritional support was initiated via the parenteral route due to enteral feeding intolerance. Caloric intake was gradually increased to 60 kcal/kg/day during the recovery phase to meet the markedly elevated metabolic demands associated with severe tetanus.

On ICU Day 22, the patient’s clinical improvement permitted discontinuation of mechanical ventilation, with successful transition to supplemental oxygen via a nebulizer mask. Analgesia and sedation were maintained with intravenous infusions of remifentanil and dexmedetomidine. On ICU Day 28, the patient achieved complete resolution of tetanic spasms, full consciousness with ability to follow complex commands, successful weaning from mechanical ventilation, and a passing grade (Grade I) on the formal Water Swallow Test, indicating safe swallowing. Following comprehensive multidisciplinary assessment, the patient was transferred to a general ward for continued rehabilitation in a hemodynamically and respiratory stable condition.

### 2.6. Clinical Course and Outcome

Spasm frequency declined progressively throughout the ICU course: from a peak of 26 episodes/day between ICU Days 9–12, to 20 episodes/day by ICU Day 13, and further to 6 episodes/day by ICU Day 21, allowing continued weaning of sedation. The autonomic dysfunction gradually resolved by ICU Day 13, coinciding with the weaning of norepinephrine and the progressive decline in spasm frequency. Thereafter, hemodynamic parameters remained stable without further vasopressor support.

On ICU Day 22, the patient’s clinical improvement permitted discontinuation of mechanical ventilation, with successful transition to supplemental oxygen via a nebulizer mask. Analgesia and sedation were maintained with intravenous infusions of remifentanil and dexmedetomidine. Parenteral nutrition was continued at 60 kcal/kg/day. Structured mobilization protocols were initiated. During this phase, the patient developed delirium, prompting implementation of the ABCDEF bundle for delirium mitigation.

By ICU Day 28, the patient had achieved significant clinical milestones: Complete resolution of tetanic spasms and convulsions; full consciousness with ability to follow complex commands; successful weaning from mechanical ventilation with independent respiration via a tracheostomy mask with oxygen at 2 L/min through a heat and moisture exchanger; passing a formal Water Swallow Test (Grade I), indicating safe swallowing; and demonstrated active mobilization of all limbs and trunk.

Following a comprehensive multidisciplinary team assessment, the patient was deemed stable for transfer to a general ward to continue rehabilitation. We administered the tetanus‐diphtheria (Td) vaccine to the patient on ICU Day 28 (the day of discharge from the ICU). A second Td booster is scheduled for 2 months postdischarge per standard guidelines. At the time of transfer, the patient was hemodynamically and respiratory stable, with full resolution of tetanic symptoms. A timeline of clinical events, investigations, and key therapeutic interventions from admission to long‐term follow‐up is summarized in Table 1.

**TABLE 1:** Timeline of clinical course and management.

Date/time point	Key clinical events	Therapeutic interventions
May 12, 2025	Onset of cough and sputum production (15‐day history). Developed acute trismus (lockjaw) and dysphagia (inability to eat/drink).	Presented to local hospital. Initial evaluation suggested cerebral infarction. Started on neuroprotective agents and conservative stroke management.
May 18, 2025	Developed generalized spasms and respiratory distress.	Transferred to tertiary hospital for further evaluation. Diagnostic workup: Head CT: Multiple lacunar infarcts, leukoaraiosis. Chest CT: Mild left pneumonia, bilateral nodules, left pleural effusion. Brain MRI: Confirmed lacunar infarcts, ruled out acute stroke. Intervention: Endotracheal intubation and mechanical ventilation initiated.
May 18–May 20, 2025	Suspected encephalitis. Patient remained undiagnosed. Neurological consultation obtained.	Consulted by ICU team from our hospital. Clinical findings during consultation: Trismus, risus sardonicus, opisthotonos, stimulus‐triggered spasms, preserved consciousness.
ICU Day 1	Diagnosis of Severe Tetanus (Ablett Grade IV) confirmed, based on severe generalized spasms, respiratory failure requiring mechanical ventilation, and autonomic dysfunction requiring vasopressor support. Transferred to our ICU.	Tracheostomy performed. Mechanical ventilation (VC‐SIMV mode). Immediate treatment: HTIG (3000 IU, IV) administered. Metronidazole (500 mg q8h, IV) initiated (planned for 10‐day course). Deep sedation/analgesia initiated (remifentanil, midazolam, propofol, dexmedetomidine). Comprehensive full‐body examination (scalp, oral cavity, nail beds, interdigital spaces, soles, perianal region): no active wound identified. Only remote facial scarring from burns > 20 years prior noted.
ICU Day 3–8	High frequency of spasms (up to 16 episodes/24 h). Autonomic dysfunction: systolic BP 80–190 mmHg, tachycardia (HR 120–150 bpm), diaphoresis; fluctuations triggered by spasms and external stimuli.	Norepinephrine continuous infusion initiated (0.05 μg/kg/min, titrated to max 0.3 μg/kg/min, weaned off by ICU Day 13). Aggressive fluid/electrolyte and parenteral nutritional support.
ICU Day 9–12	Transient exacerbation of spasms (peak at 26 episodes/day).	Sedative/analgesic infusion rates temporarily increased. Spasm frequency gradually declined thereafter.
ICU Day 13	Spasm frequency decreased to 20 episodes/day.	Continued weaning of sedation.
ICU Day 21	Spasm frequency further decreased to 6 episodes/day.	Continued supportive care and early mobilization.
ICU Day 22	Significant clinical improvement.	Mechanical ventilation discontinued. Successfully transitioned to supplemental oxygen via nebulizer mask. Developed delirium, prompting ABCDEF bundle implementation.
ICU Day 28	Complete resolution of tetanic spasms. Full consciousness, able to follow complex commands. Passed Water Swallow Test (Grade I), indicating safe swallowing. Active mobilization of all limbs and trunk.	Td vaccine administered. Transferred to general ward for rehabilitation. Second Td booster scheduled for 2 months postdischarge per standard guidelines.

## 3. Discussion

### 3.1. Epidemiological Context and Prevention Strategies in China

The incidence of tetanus in China has decreased markedly over recent decades, largely attributable to the Expanded Program on Immunization (EPI). The current national immunization schedule recommends primary vaccination with the diphtheria‐tetanus‐pertussis (DTaP) vaccine at 2, 4, and 6 months of age, followed by booster doses at 18 months and 6 years of age. Despite this framework, booster coverage among adults and older adults remains suboptimal. A considerable proportion of the elderly population lack protective antibody titers, a consequence of waning vaccine‐induced immunity compounded by the absence of a national booster program targeting this age group.

Although nationwide incidence data specific to adults are not systematically reported, tetanus cases continue to emerge, with a predilection for rural areas and agricultural workers. However, the patient in the present case was a retired factory worker residing in an urban area, with a history of agricultural work prior to retirement. According to Chinese clinical practice guidelines, HTIG is recommended at a prophylactic dose of 250–500 IU for contaminated wounds, whereas therapeutic dosing for established tetanus is typically 3000–6000 IU, administered intramuscularly or intravenously, consistent with the regimen employed in our case. Importantly, patients recovering from tetanus should receive active immunization with a tetanus‐containing vaccine (e.g., Td) prior to discharge, as natural infection does not confer durable protective immunity.

### 3.2. Diagnostic Challenges in Tetanus Without Identifiable Portal of Entry

This case illustrates the diagnostic difficulties posed by tetanus when an acute, identifiable wound is absent. The patient’s initial presentation with trismus and dysphagia led to neurological consultation and misdiagnosis as cerebral infarction. This pattern of misdirection is not uncommon, when patients present with trismus and dysarthria; Healthcare providers frequently suspect a primary neurological disorder, and pre‐examination triage often directs patients directly to neurology departments. In this case, extensive ancillary investigations within the neurology department failed to identify a definitive cause for the spasms, resulting in diagnostic uncertainty and a delay in reaching the correct diagnosis.

The absence of an identifiable portal of entry further complicated the diagnosis. Tetanus occurs without an obvious wound or identifiable portal of entry in a substantial proportion of cases, particularly in older adults. Patients may not recall minor injuries, and healed or small wounds can make the portal of entry difficult to identify on routine examination. In this case, a meticulous full‐body examination failed to identify any active wound, ulcer, or recent injury. The only identifiable skin abnormality was facial scarring from burns sustained more than 20 years prior. It is biologically implausible to attribute the current episode of tetanus to a wound sustained more than two decades earlier. We, therefore, consider this remote scar an incidental examination finding and conclude that the portal of entry remained unidentified. More likely explanations include an unidentified minor injury, a healed wound that was no longer visible, or inoculation through a chronic skin condition.

### 3.3. Differentiating Tetanus From Other Neurological Conditions

Key clinical features that support the diagnosis of tetanus, rather than encephalitis with generalized tonic–clonic seizures, include the following observations, which should be considered as helpful clinical clues rather than absolute discriminators.

Spasm or convulsion characteristics: Tetanus spasms are often stimulus‐sensitive (triggered by noise, light, suction, or touch), manifesting as trismus/lockjaw, facial grimacing (risus sardonicus), rigidity (opisthotonos), and characteristic posturing (e.g., clenched fists and flexed arms). In contrast, encephalitis seizures are typically spontaneous, generalized, convulsive episodes, often followed by postictal drowsiness. However, encephalitis may also present with focal or generalized seizures and variable impairment of consciousness. Notably, patients with tetanus typically remain fully conscious during spasms, though communication may be impaired, with consciousness returning to baseline between episodes. In critically ill patients receiving sedation, however, consciousness may be altered, and this feature should be interpreted with caution.

Temperature profile: Tetanus patients may exhibit fever primarily during spasms, with temperature normalizing afterward. Encephalitis usually presents with persistent high‐grade fever unrelated to muscular activity.

Consciousness: The preservation of consciousness during spasms is a highly characteristic feature of tetanus and an important differentiating feature from encephalitis seizures, which typically involve loss of consciousness and postictal depression. Nevertheless, sedated ICU patients with tetanus may have altered consciousness due to medications, respiratory failure, or other complications.

Other conditions in the differential diagnosis of acute trismus and dysphagia in older adults include drug‐induced dystonia, dental or deep neck infections, temporomandibular disorders, hypocalcemia, neuroleptic malignant syndrome, and toxicologic causes. A thorough history, medication review, and physical examination are essential to exclude these entities. The diagnosis of tetanus depends on the overall clinical pattern, including trismus, generalized rigidity, characteristic posturing, stimulus‐sensitive spasms, and the evaluation of reasonable alternative diagnoses.

### 3.4. Tetanus in Older Adults

Older adults are at a particular risk for tetanus due to waning immunity from childhood vaccination and lack of booster doses. In China, the recommended tetanus vaccination schedule includes primary vaccination in childhood with diphtheria‐tetanus‐pertussis (DTP) vaccine, followed by booster doses at school entry and in early adulthood. However, booster vaccination coverage in older adults is often suboptimal, leaving this population vulnerable to tetanus. The incidence of tetanus in China has declined substantially with improved vaccination coverage, but cases continue to occur, particularly in rural areas and among older adults with agricultural exposure. In the present case, the patient was a retired factory worker with a history of agricultural work, which may have contributed to past environmental exposure.

Recent data from Japan demonstrate that older adults with tetanus often require prolonged ICU stays and have significant functional impairment at discharge [9]. This case is consistent with these findings, as the patient required 28 days of ICU care and intensive rehabilitation. The functional prognosis of older adults with tetanus depends on early recognition, prompt administration of antitoxin, aggressive supportive care, and comprehensive rehabilitation.

### 3.5. Occult or Unidentified Portal of Entry

The phenomenon of tetanus occurring without an identifiable portal of entry is well recognized in the literature, with a substantial proportion of patients unable to recall any recent injury or wound. Several potential explanations exist for this observation, including minor injuries that went unnoticed or were subsequently forgotten, healed wounds that are no longer visible on physical examination, chronic skin conditions such as ulcers or fissures that may serve as cryptic portals, contaminated injection sites, dental procedures or oral mucosal disruptions, and even gastrointestinal or other mucosal routes of entry. In the present case, although the patient had a history of agricultural work prior to retirement, no identifiable portal of entry was found on thorough examination, and the remote facial scar was considered an incidental finding rather than a causal factor.

We acknowledge the limitations inherent to a single case report, including its limited generalizability. The absence of an identified portal of entry and the lack of laboratory confirmation (e.g., via wound culture or detection of tetanus toxin) represent major limitations. While the diagnosis was confidently made on clinical grounds, we cannot definitively prove that the remote facial scar was not the entry point, though it appears biologically unlikely.

## 4. Conclusion

This case emphasizes that severe generalized tetanus should remain in the differential diagnosis of older adults presenting with acute trismus, dysphagia, muscle rigidity, or stimulus‐triggered spasms, even when recent trauma is not recalled and no active wound is visible. The absence of an identifiable portal of entry should not delay diagnosis or treatment. Early recognition of the highly characteristic stimulus‐sensitive spasms in a conscious patient is critical to prevent diagnostic delays and the initiation of misguided treatment pathways.

Clinicians should obtain a thorough exposure history, including occupation, living environment, and activities, to assess tetanus risk. Repeated full‐body examinations should be performed to identify potential portals of entry, including the soles, toes, nail folds, oral cavity, perianal region, and old scar areas. Complete tetanus management requires early administration of human tetanus immunoglobulin, antimicrobial therapy, intensive sedation and analgesia, respiratory support, and active immunization during recovery.

Strict adherence to established tetanus management protocols, encompassing supportive care and antitoxin administration, is paramount for survival and functional recovery. This report aims to raise clinician awareness of these atypical presentations to facilitate prompt, life‐saving intervention.

## Author Contributions

Yanwei Rao: conceptualization, funding acquisition, writing–original draft, and writing–review and editing; Wei He, Yang Yu, Yicheng Wang, and Yuan Yuan: writing–original draft and writing–review and editing; Yongjie Wang: funding acquisition, writing–original draft, and writing–review and editing.

## Funding

This work was supported by the Jilin Provincial Health and Family Planning Commission (Grant Numbers: 2023LC042) and Jilin Province Clinical Medical Research Center for Emergency & Critical Care Medicine (Grant numbers: YDZJ202502CXJD064).

## Disclosure

All authors have read and approved the final version of the manuscript.

## Ethics Statement

This case report does not constitute research involving human subjects as defined by Chinese regulations and, therefore, did not require institutional review board approval. The patient’s legal representative provided written informed consent for publication of the clinical history, laboratory data, and anonymized radiological images. A copy of the signed consent form is available from the corresponding author on request.

## Consent

Please see the Ethics Statement.

## Conflicts of Interest

The authors declare no conflicts of interest.

## Supporting Information

Additional supporting information can be found online in the Supporting Information section.

## Supporting information


**Supporting Information** Care Checklist.

## Data Availability

All data generated or analyzed during this case report are included within this published article. Additional anonymized data supporting the findings are available from the corresponding author upon reasonable request, subject to applicable Swissdata‐protection regulations.
